# DIE-RNA: A Reproducible Strategy for the Digestion of Normal and Injured Pancreas, Isolation of Pancreatic Cells from Genetically Engineered Mouse Models and Extraction of High Quality RNA

**DOI:** 10.3389/fphys.2018.00129

**Published:** 2018-02-27

**Authors:** Mohamad Assi, Nicolas Dauguet, Patrick Jacquemin

**Affiliations:** ^1^de Duve Institute, Université catholique de Louvain, Brussels, Belgium; ^2^de Duve Institute, Flow Cytometry and Cell Sorting Facility (CYTF), Brussels, Belgium

**Keywords:** pancreatitis, cell isolation, flow cytometry, inflammation, pancreas, RNA quality control

## Abstract

The isolation of ribonucleic acid (RNA) suitable for gene expression studies is challenging in the pancreas, due to its high ribonuclease activity. This is even more complicated during pancreatitis, a condition associated with inflammation and fibrosis. Our aim was to implement a time-effective and reproducible protocol to isolate high quality RNA from specific pancreatic cell subtypes, in normal and inflammatory conditions. We used two genetically engineered mouse models (GEMM), Ela-CreER/YFP and Sox9-CreER/YFP, to isolate acinar and ductal cells, respectively. To induce pancreatitis, mice received a caerulein treatment (125 μg/kg) for 8 and 72 h. We alternatively used EGTA and calcium buffers that contain collagenase P (0.6 mg/mL) to rapidly digest the pancreas into individual cells. Most of the cells from normal and injured pancreas were single-dissociated, exhibited a round morphology and did not incorporate trypan blue dye. Cell suspensions from Ela- and Sox9-CreER/YFP pancreas were then sorted by flow cytometry to isolate the YFP-positive acinar and ductal cells, respectively. Sorted cells kept a round shape and emitted fluorescence detected by the 38 HE green fluorescence filter. RNA was isolated by column-based purification approach. The RNA integrity number (RIN) was high in sorted acinar cell fractions treated with or without caerulein (8.6 ± 0.17 and 8.4 ± 0.09, respectively), compared to the whole pancreas fraction (4.8 ± 1.1). Given the low number of sorted ductal cells, the RIN value was slightly lower compared to acini (7.4 ± 0.4). Quantitative-PCR experiments indicated that sorted acinar and ductal cells express the specific acinar and ductal markers, respectively. Additionally, RNA preparations from caerulein-treated acinar cells were free from significant contamination with immune cell RNA. We thus validated the DIE (Digestion, Isolation, and Extraction)-RNA tool as a reproducible and efficient protocol to isolate pure acinar and ductal cells *in vivo* and to extract high quality RNA from these cells.

## Introduction

Acute pancreatitis is among the most prevalent diseases that affect the pancreas and is a main driver for pancreatic cancer development (Yadav and Lowenfels, 2013). Acinar cells, which represent 80% of the pancreas, are mainly affected by pancreatitis (Reichert et al., 2016). In the presence of inflammation, acinar cells undergo acinar-to-ductal metaplasia, a pre-requisite for the development of pancreatic neoplasia and, possibly, pancreatic cancer in a later stage (Strobel et al., 2007; Fendrich et al., 2008; Prévot et al., 2012). However, the pathophysiology of metaplasia initiation is still incompletely understood. In this context, preclinical models of pancreatitis are of particular interest to get more insights into the underlying molecular mechanisms. Accordingly, significant advances could be made by profiling gene expression and applying deep molecular biology approaches on purely isolated acinar cells from injured pancreas.

The extraction of high quality ribonucleic acid (RNA) suitable for reproducible gene expression experiments and RNA sequencing is difficult to achieve in pancreas, compared with other organs such as skeletal muscle and liver. This is due to the exocrine function of the pancreas that produces excessive amounts of ribonucleases, as well as deoxyribonucleases and proteases. A mouse pancreas contains ~75 mg of ribonucleases, which are partly released during dissection and RNA preparation procedures. This contributes to tissue autolysis and the low quality of the resulting RNA preparations (Lenstra and Beintema, 1979). Moreover, in the presence of pancreatitis, a further decrease in RNA quality occurs. Therefore, the high enzymatic activity of the pancreas in normal and pathological conditions, constitute a serious barrier against the application of standard molecular biology approaches in pancreatology.

Our laboratory and others have previously developed specific protocols or optimized existing ones to isolate intact RNA from the pancreas. Examples of strategies adopted involve: immersion of the pancreas in RNAlater for 24 h at −80°C, ductal infusion with a pH-controlled sulfate salt solution and incubation at room temperature (RT) for 24-to-72 h, rapid storage of the pancreas in liquid nitrogen upon dissection, and use of a high phenol/guanidine-to-tissue ratio (Mullin et al., 2006; Kiba et al., 2007; Azevedo-Pouly et al., 2014; Augereau et al., 2016). Although these different methods yield satisfactory RNA quality, they still present two major limitations. *Firstly*, these methods only allow RNA extraction from whole pancreatic tissue and are not suitable for the preparation of RNA from a given pancreatic cell type. *Secondly*, their application in conditions evoking pancreatitis has not been previously tested and may probably lead to the collection of poor quality RNA.

In the present work, we describe an effective strategy to obtain RNA from specific pancreatic cell types that combines transgenic technology, flow cytometry, and cellular and molecular biology techniques. This protocol is easily undertaken, time-effective, and reproducible, enabling the isolation of pure acinar cells from mouse pancreas in both normal and acute inflammation conditions for extraction of high quality RNA with an average RNA integrity number (RIN) of 8.5. This method is also applicable to ductal cells for the isolation of RNA suitable for sensitive molecular biology applications. We thus validated the DIE (Digestion, Isolation, and Extraction)-RNA tool as a reproducible and efficient protocol to isolate pure acinar and ductal cells *in vivo* and subsequently extract high quality RNA.

## Materials and equipment

### Animals

All procedures described below were performed with the approval of the animal welfare committee of the University of Louvain Medical School. Mice received humane care according to the criteria listed by the National Academy of Sciences. Mice used in this study were mainly maintained in an enriched CD1 background. Elastase-CreER/ROSA26^Yellow Fluoresence Protein (YFP)/+^ (Ela-CreER/YFP) and Sox9-CreER/YFP were obtained after breeding Ela- or Sox9-CreER males with ROSA26^YFP/YFP^ females. Ela-CreER/YFP and Sox9-CreER/YFP were used to isolate acinar and ductal cells, respectively.

**Figure 1:**
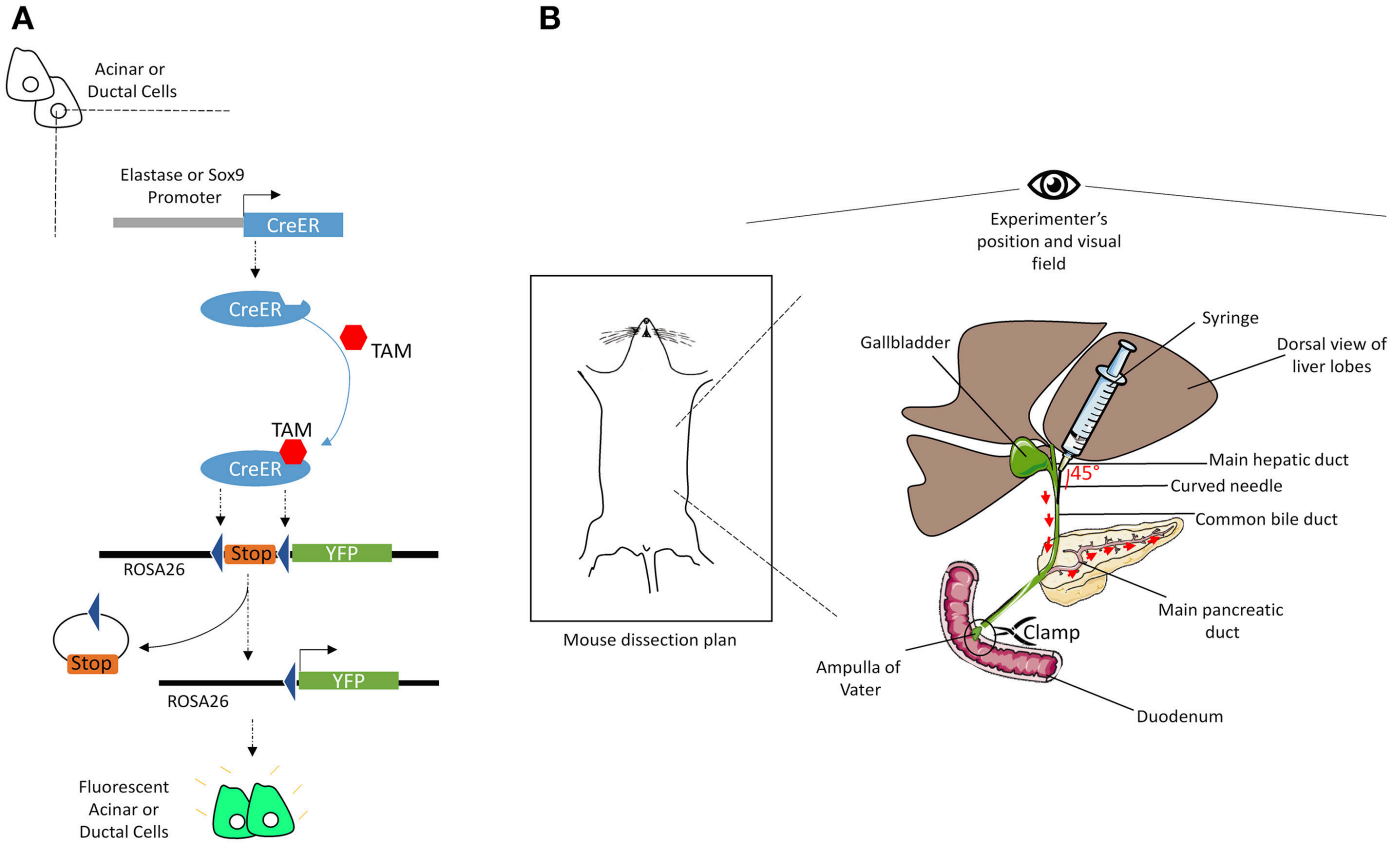
Illustrations of the mechanisms of CreER recombination and of common bile duct injection. **(A)** The elastase or Sox9 promoter located upstream of the CreER gene allows specific CreER expression in acinar or ductal cells, respectively. The presence of TAM induces Cre-mediated recombination, through its specific interaction with the ligand binding domain of the estrogen receptor (ER) coupled to the Cre. Activated CreER deletes the stop cassette inserted between the two loxP sites in the ROSA26 locus, upstream of the gene coding for YFP, *via* homologous recombination. Thus, YFP is specifically expressed in acinar or ductal cells. **(B)** After dissection, mouse's head should be placed face up the experimenter. Using two straight forceps flip the liver lobes so that the common bile duct becomes visible, from the liver to the duodenum. Clamp the intersection point of the duct with the duodenum (Ampulla of Vater). Then, delicately insert a curved needle (30 gauge) in the duct and inject 3-to-5 mL of cold Col-EGTA solution. The pancreas will blow and take a white appearance. The detailed technical procedure is provided in the text.

Four to 12-week-old mice were injected subcutaneously with 100 μL tamoxifen (TAM) (30 mg/mL, in corn oil) combined with a gavage of 4-hydroxytamoxifen (0.3 mg/mL, in corn oil) once a day and every other day over 5 days. Figure 1A illustrates the mechanism by which TAM induces the expression of YFP specifically in acinar or ductal cells. To induce acute pancreatitis, mice received seven intra-peritoneal injections of caerulein (125 μg/kg) per day; either for 1 day or for 2 days separated by 1 day of rest (less caerulein may be necessary depending on the genetic background of animals). Mice were sacrificed either at day 1 after the completion of the first series of injections or at day 4 following the second series of injections. The protocol was optimal when the weight of the mouse was between 20 and 25 g.

### Reagents and enzymes

- Agilent RNA 6000 Nano kit (5067-1512, Agilent Genomics, USA)
- Bovine serum albumin (BSA) (160069 MP, Merck, Belgium)
- Caerulein (C9026, Merck, Belgium)
- Collagenase P 500 mg (1121386500, Merck, Belgium)
- Deoxyribonucleotide (dNTP) (03622614001, Merck, Belgium)
- Dulbecco's phosphate-buffered saline (DPBS) (BE17-512F, Lonza, Belgium)
- High-capacity complementary deoxyribonucleic acid (cDNA) reverse transcription kit (4368814, Life technology Europe, Belgium)
- KAPA SYBR FAST (KK4608, Merck, Belgium)
- Nuclease-Free water (Promega, Netherlands)
- Prepared solutions listed in Table 1
- Pure Ethanol 100% (UN1170, VWR Chemicals, Belgium)
- RNAqueous-Micro total RNA isolation kit (AM1931, Fisher Scientific, Belgium)
- RNaseOut (10777019, Life technology Europe, Belgium)
- Tamoxifen (T5648, Merck, Belgium)
- Trypan blue (T8154, Merck, Belgium)

**Table 1:** Composition of EGTA- and calcium-buffers.

**References**	**Stock solutions**	**EGTA-buffer composition**	**Calcium-buffer composition**
194848, Merck, Belgium	NaCl (1 M)	125 mM	123 mM
C7902, Merck, Belgium	CaCl_2_ (1 M)	–	1.8 mM
1058860500, Merck, Belgium	MgSO_4_ (1 M)	0.8 mM	0.8 mM
1049360500, Merck, Belgium	KCl (3 M)	5.4 mM	5.4 mM
65791000, Merck, Belgium	NaH_2_PO_4_ (1 M)	1 mM	1 mM
1707, Merck, Belgium	NaHCO_3_ (1 M)	4.2 mM	4.2 mM
H3375-100G, Merck, Belgium	HEPES (1 M)	10 mM	10 mM
Fisher Scientific, Belgium	Glucose (1 M)	5.6 mM	5.6 mM
E4378-100G, Merck Belgium	EGTA (0.1 M)	7.4 mM	–

### Equipment

- 0.22 μm-filter (430758, Fisher Scientific, Belgium)
- 30-gauge needle (30400, BD Biosciences, Belgium)
- 40 μm-cell strainer (431750, Fisher Scientific, Belgium)
- 0.2, 0.5, and 1.5 mL-Sterile Eppendorf tubes (Eppendorf, Belgium)
- 15 and 50 mL-Polypropylene Falcon tubes (Fisher Scientific, Belgium)
- 250 and 500 mL-Pyrex bottles (Fisher Scientific, Belgium)
- 96-well PCR plates (HSP9655, Bio-Rad, Belgium)
- Automated cell counter (TC20, Bio-Rad, Belgium)
- Cell sorter machine with water bath (FACS ARIAIII, BD Biosciences, Belgium)
- CFX96 real-time PCR machine (C1000, Bio-Rad, Belgium)
- Fluorescence microscope (Axiovert 200, Zeiss, Belgium)
- Incubator at 37°C with a rotating plate (Eppendorf, Belgium)
- Magnetic bar and agitator
- Nanodrop spectrophotometer (ND-1000, Isogen, Belgium)
- Nuclease-free tips and pipettes (Westburg, Netherlands)
- Optical microscope (Axiovert 40C, Zeiss, Belgium)
- Pyrex beaker (Fisher Scientific, Belgium)
- RNA BioAnalyzer system (Agilent 2100, Agilent Genomics, Belgium)
- Sterile dissection tools (i.e., scissors, forceps, and clamps) (Fine Science Tools, Germany)
- Sterile Petri dishes (Fisher Scientific, Belgium)
- Thermo-cycler machine (T100, Bio-Rad, Belgium)

### Reagent set-up

► Prepare stock solutions listed in Table 1, by dissolving the required amount of powder in distilled water.
► Autoclave all solutions (30 min, 120°C) and pass them through a 0.22 μm-filter. Solutions can then be stored at room temperature (RT) for up to 6 months or until the formation of crystal precipitates.
► The day before the experiment, prepare EGTA- and calcium-buffers separately in Pyrex beakers (see Table 1).
► Place EGTA- and calcium-buffers on an agitator and add BSA powder (0.5%). The complete dissolution may take 20 min.
► Adjust the pH of both buffers to 7.4 and filter them through a 0.22 μm-filter. Store buffers at 4°C overnight (it is recommended to always use fresh buffers).
► Dispense collagenase P powder into aliquots of 12 mg in Falcon polypropylene tubes (50 mL). Two aliquots are needed per mouse.
► The day of experiment, dissolve one aliquot of collagenase P in 20 mL of EGTA-buffer and another in 20 mL of calcium-buffer, to obtain 0.6 mg/mL collagenase-EGTA (Col-EGTA) and collagenase-calcium (Col-Ca^2+^) buffers, respectively.
► Keep Col-EGTA and Col-Ca^2+^ buffers on ice, until proceeding to dissection.
► In a 50 mL-Falcon tube, prepare a solution of EGTA-buffer containing RNaseOut (20 U/mL) and store at 4°C. This solution will be called “sorting buffer” in the protocol.

### Equipment set-up

► Before proceeding to dissection, turn on the incubator to stabilize the temperature at 37°C.
► Take 5 mL of Col-EGTA buffer with a 10 mL-syringe.
► Draw through a 30-gauge needle into the syringe.
► Curve the needle carefully with your finger to obtain an angle of ~45°. This will make injection easier.
► Close the plug and keep the syringe on ice.

## Stepwise procedure

### Common bile duct injection • TIME 10 min

The Figure 1B shows a scheme of the main steps of injection and provides an overview of the procedure before application.

A stereoscopic microscope, although not essential, can help to perform this step.

**Figure 2:**
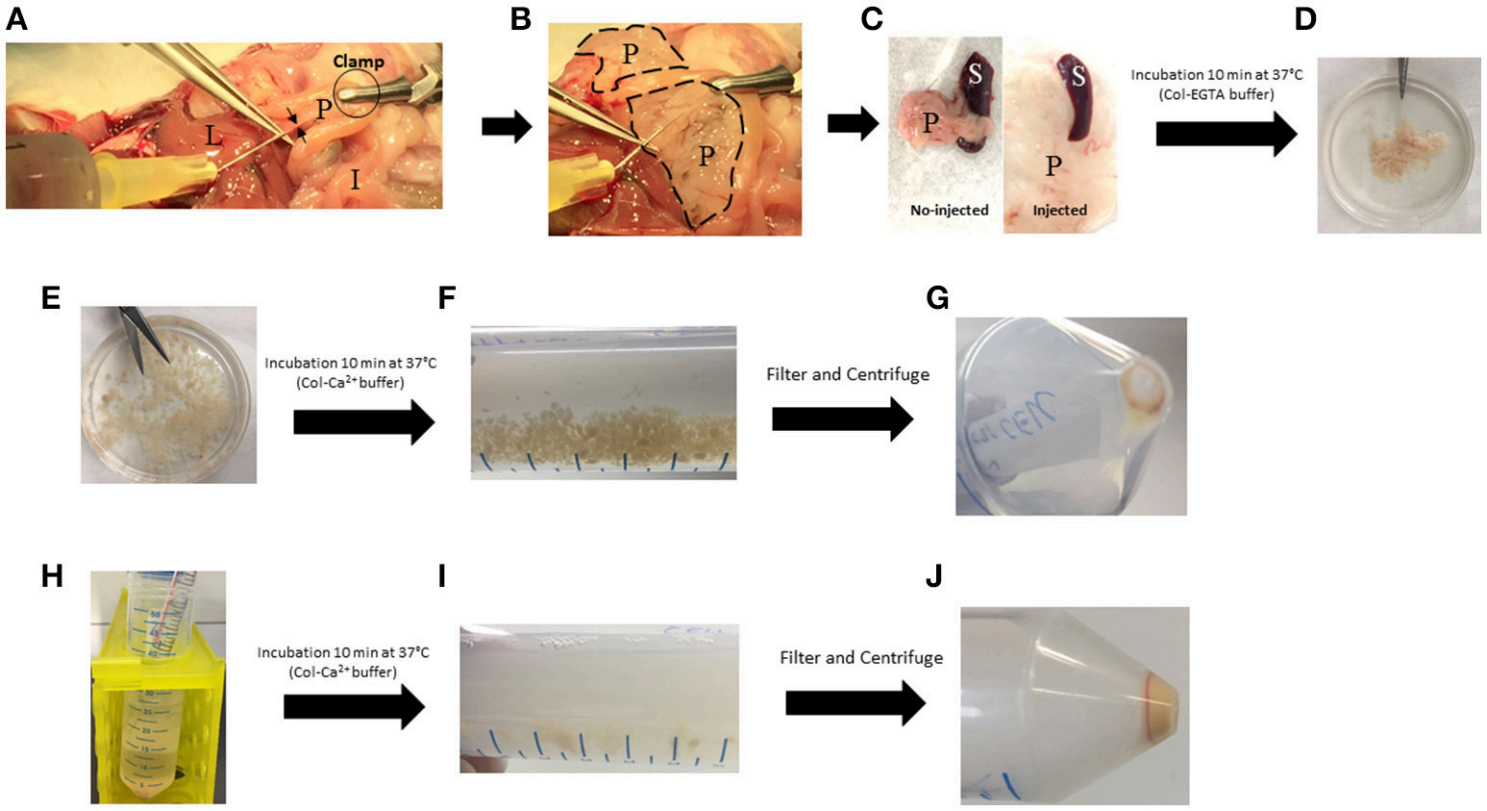
Key steps of the pancreas digestion protocol. **(A)** Injection of cold Col-EGTA buffer in the common bile duct (black arrows). **(B)** Pancreas (dotted lines) blows and becomes white. **(C)** Comparison between injected and no-injected pancreas. **(D)** Pancreas takes a floppy texture. **(E)** Pancreas is cut into small pieces. **(F)** Pancreas is partially digested with Col-Ca^2+^ buffer and small pieces are still present. **(G)** First pellet of dissociated cells that must be kept on ice. **(H)** Col-Ca^2+^ buffer is setback on remaining undigested pancreas. **(I)** Digestion solution takes a milky appearance. **(J)** Final pellet of cell that must be kept on ice. I, Intestine; L, Liver; P, Pancreas; S, Spleen.

1. Kill the mouse by cervical dislocation and directly decapitate the animal using standard sharp scissors.
2. Make an incision at the bottom of the abdomen (near the genital parts, for a male mouse, or the equivalent place for a female mouse), then open the thoracic cavity and remove the ribcage.
3. Turn over the liver lobes so you can have a dorsal view of them. The common bile duct will appear as a white tube coming out of the liver to the duodenum.
4. Hold the duodenum using a ring forceps and block the intersection point (resembling a white spot) between the common duct and the duodenum with a clamp.
5. Position the clamp near the genital region to create tension on the duct and facilitate the injection.
6. Take straight forceps with fine tips in one hand and a syringe with a curved needle containing EGTA-buffer in the other hand.
7. Place the forceps gently under the common bile duct to offer support and avoid duct shredding during injection.
8. Insert the needle delicately in the duct and slowly inject 3-to-4 mL of EGTA-buffer. The pancreas distends and takes on a white color (Figures 2A,B).
9. After injection, hold the spleen with straight forceps and remove the pancreas and spleen away from the stomach and duodenum.

### Pancreas digestion •TIME 60 min

A detailed graphical illustration of this step can be found in supplementary data (Figure S1).

1. In a sterile Petri dish, remove the spleen and contaminating mesenteric tissue (Figure 2C). Put the pancreas in a 50 mL-polypropylene Falcon (Tube 1) containing 10 mL of cold fresh Col-EGTA medium.
2. Incubate Tube 1 at 37°C for 10 min with continuous shaking (200 rotations/min). Place the tube horizontally to allow better dissociation.
3. After incubation, put the pancreas in a sterile Petri dish and cut it into small pieces of ~2 mm with scissors (Figures 2D,E).
4. Put the pancreas back into Tube 1 and wash rapidly twice with 20 mL of sterile DPBS to remove floating adipose tissue and EGTA traces. Allow the tube to stand so pancreatic tissue can sediment.
5. Add 20 mL of fresh Col-Ca^2+^ buffer and incubate at 37°C for 10 min with continuous shaking (200 rotations/min). At the end of this step, pancreas fragments are still present in the solution (Figure 2F). ▴CRITICAL Use fresh Ca^2+^-buffer (prepared the day before the experiment) (Table 2).
6. Filter the solution through a 40 μm-cell strainer into a new Falcon (Tube 2) and centrifuge at (500 g × 3 min at 4°C).
7. Return the supernatant into Tube 1 with the remaining undigested pancreas fragments and keep the pellet of dissociated cells in Tube 2 on ice. Slowly put 1 mL DPBS on the pellet and rotate the Falcon gently, without disrupting the pellet. Remove DPBS, then resuspend the pellet with an additional 500 μl of fresh DPBS and keep on ice. Continue digestion at 37°C for 10 min with shaking (200 rotations/min) (Figures 2G,H).
8. At the end of step 7, pass the solution in Tube 1 through a 40 μm-filter into Tube 2 and centrifuge at (500 g × 3 min at 4°C) (Figures 2I,J).
9. Wash the pellet once to remove residual collagenase by suspending it with 10 mL DPBS. Centrifuge at (500 g × 3 min at 4°C).
10. Add 20 mL of EGTA-buffer and suspend the pellet by pipetting up and down with a 10 mL-pipette. This step is optional, but results in better dissociation of the cells.
11. Check using an optical microscope that the cells are not damaged and are well-dissociated.
12. Centrifuge at (500 g × 3 min at 4°C), and resuspend the pellet in 1 mL of sorting buffer.
13. ▴CRITICAL Keep the cells on ice until sorting by flow cytometry.

**Table 2:** Troubleshooting and practical advice.

**Step**	**Potential problem**	**Cause**	**Solution**
Common bile duct injection	Uncomplete injection of the pancreas	Duct perforation	Continue digestion according to the protocol (you should obtain a good yield)
Pancreas digestion	Partial digestion of caerulein-treated pancreas	Fibrosis may make the tissue more resistant	Incubate an additional 5-to-10 min with Col-Ca^2+^ buffer at 37°C
	Cell mortality	High collagenase activity/Prolonged incubation	Check collagenase batch/reduce time of digestion to 5 min during the second incubation with Col-Ca^2+^ buffer
Flow Cytometry	Low number of sorted cells	False gating parameters/Naturally low number of cells	Ensure that the gate covers all YFP-positive events/sort two pancreas digested separately in one tube
	Stuffy nozzle	High number of small debris/DNA rings formation	Use fresh buffers
	YFP-positive cells shift toward more PI staining	Cell damage during sorting	Reduce pressure and drop frequency
RNA extraction and quality	Some samples have remarkably high RNA contamination	Filter may flip over	Avoid touching the filter with tips
	Undetectable RNA bands	RNA is not well-mixed with the gel	Vortex the microfluid chip for 1 min

### Cells sorting by flow cytometry •TIME 60 min

1. Pass the cells suspension through a 35 μm-filter into a polystyrene sorting tube and add propidium iodide (PI) (2 μg/mL).
2. Vortex briefly and load the tube into the cell sorter.
3. Open two dot-plots: the first dot-plot shows the forward scatter (FSC) and side scatter (SSC) and the second dot-plot gives the fluorescence channels for YFP (excitation 488 nm, collection with Band-Pass filter 530/30) and PI detection (excitation 561 nm, collection with Band-Pass filter 582/15).
4. Before the first sorting, we recommend the use of a non-YFP mouse to determine the level of auto-fluorescence and thus to easily identify the viable YFP-positive population in the Ela- or Sox9-CreER/YFP mice (and/or in the other fluorescent protein-bearing animal models used).
5. Load the next tube from Ela- or Sox9-CreER/YFP pancreas and set a first gate to eliminate most of the debris in the FSC/SSC dot-plot. Then, perform doublet discrimination on FSC (FSC-A/FSC-W) and SSC (SSC-A/SSC-W). Ductal cells form a clearly distinct population, compared to acinar cells that tend to be more widespread. Finally, create a sorting gate around the YFP-positive cells, which should also be PI-negative.
6. For sorting, we used the following parameters: nozzle 85 μm, pressure 45 psi, drop frequency 62 kHz and sort precision 16-32-0.
7. Sort your cells into a 15 mL-Falcon tube that contains 3 mL of sorting buffer. When the number of sorted cells reaches ~150,000, you can stop sorting and proceed to RNA extraction.
8. To analyze the efficiency of sorting, take a small volume of sorted cells and load into the machine. At least 75% of the cells should fall in the gate used previously for sorting.
9. ▴CRITICAL Perform the sorting procedure at 4°C.

### RNA extraction and quality control •TIME 80 min

This step has been performed by referring to the manufacturer's protocol (AM1931). However, some practical tips were added to adapt the protocol according to the number of sorted cells.

1. Centrifuge the tube at (500 g × 5 min at 4°C) to pellet the cells.
2. Add 100 μL of lysis buffer and pipet up and down to break the cells. If the cell number is < 50,000 cells, reduce volume of lysis buffer to 50 μL.
3. Add 50 μL of pure 100% ethanol for each 100 μL of lysis buffer and vortex briefly.
4. Put the lysis buffer/ethanol mixture onto the filter column and close the plug.
5. Centrifuge at (15,000 g × 30 s at RT).
6. Wash the filter with ethanol-based solutions: once with 180 μL of Wash 1 solution and twice with 180 μL of Wash 2/3 solution. Each washing step is followed by centrifugation at (15,000 g × 30 s at RT).
7. To eliminate residual ethanol, centrifuge once as in step (5).
8. Add 12 μL of pre-heated elution buffer to the filter and incubate 1 min at RT, then centrifuge at (15,000 g × 30 s at RT). Repeat step (8) once. If the cell number is < 50,000 cells, add 12 μL of pre-heated elution buffer to the filter and incubate 5 min at RT. Then centrifuge at (15,000 g × 30 s at RT).
9. Take a small aliquot of extracted RNA (2 μL) for quantity and quality control and store the remaining solution at −80°C.
10. Use 1 μL of extracted RNA to measure the total amount by Nano-drop spectrophotometry.
11. For quality control, a BioAnalyzer system from Agilent Genomics was used. The BioAnalyzer from Bio-Rad is very similar in terms of chip preparation and data processing.
12. Before starting, wash the channels of the BioAnalyzer with RNaseAway solution followed by a second wash with nuclease-free water.
13. Start by filtering 550 μL of gel matrix through a spin filter by centrifuging at (1500 g × 10 min at RT). Divide aliquots into 65 μL and store at 4°C for up to 1 month.
14. Add 1 μL of intercalating dye mix in 65 μL of gel matrix and vortex briefly. Centrifuge once (13 000 g × 10 min at RT).
15. Before the end of the centrifugation, heat RNA samples and RNA ladder at 65°C for 5 min.
16. Add the gel into the dedicated well on the chip and allow it to diffuse into all wells by applying a pressure with the priming station.
17. To each well, add 5 μL of RNA buffer and 1 μL of RNA sample (diluted 1:2).
18. ▴CRITICAL Vortex the chip 1 min using a horizontal vortex (Table 2).
19. Put the chip into the BioAnalyzer and start the program by choosing the “total eukaryotic RNA-Nano” option.
20. At the end of the run the results are given in the form of electropherograms and the 28S/18S ribosomal RNA (rRNA) bands can be visible on gel-like tracks.

## Anticipated results and discussion

### Alternation between Col-EGTA and Col-Ca^2+^ buffers produces a great number of live single-dissociated cells in healthy and injured pancreas

In the present paper, we fully describe a protocol that allows a rapid dissociation of adult pancreas, isolation of acinar or ductal cells and extraction of high quality RNA suitable for deep and sensitive molecular biology approaches. The composition of EGTA- and Ca^2+^- buffers used for the digestion is similar to that described by Stangé et al. for β-cells isolation (Stangé et al., 2003). However, the different steps of the digestion protocol, time and order of use of buffers, have been optimized. We tested different procedures to increase the yield of single-dissociated cells. For example, overnight digestion with cold trypsin at 4°C, as described by Li et al. (2013), produced an insufficient number of viable dissociated cells (~8 000 000 cells/pancreas) (data not shown). When we performed a collagenase (Col) digestion at 37°C with either EGTA- or Ca^2+^- buffer, throughout the protocol, we obtained either a low number of dissociated cells or a high number of cells still organized in clusters, respectively. We found it necessary to combine both EGTA- (Ca^2+^ chelator) and Ca^2+^-buffers in the protocol to obtain a high number of single-dissociated cells. This could be explained by the fact that Ca^2+^ is necessary for an optimal Col activity, while EGTA is needed to disrupt the desmosomal intercellular junctions and lead to a better dissociation of cells (Chidgey and Dawson, 2007; Tria et al., 2013). Thus, injection and incubation with a Col-EGTA buffer helps to irreversibly cleave the intercellular junctions (Neufeld, 1997). Additionally, Col at a concentration of 0.6 mg/mL will maintain a low activity in the EGTA solution, which is sufficient to tenderize the pancreas and prepare it for a rapid and efficient digestion. After the removal of EGTA, the addition of Col-Ca^2+^ buffer for a short time (not exceeding 20 min), results in complete pancreatic digestion (Figure 2).

**Figure 3:**
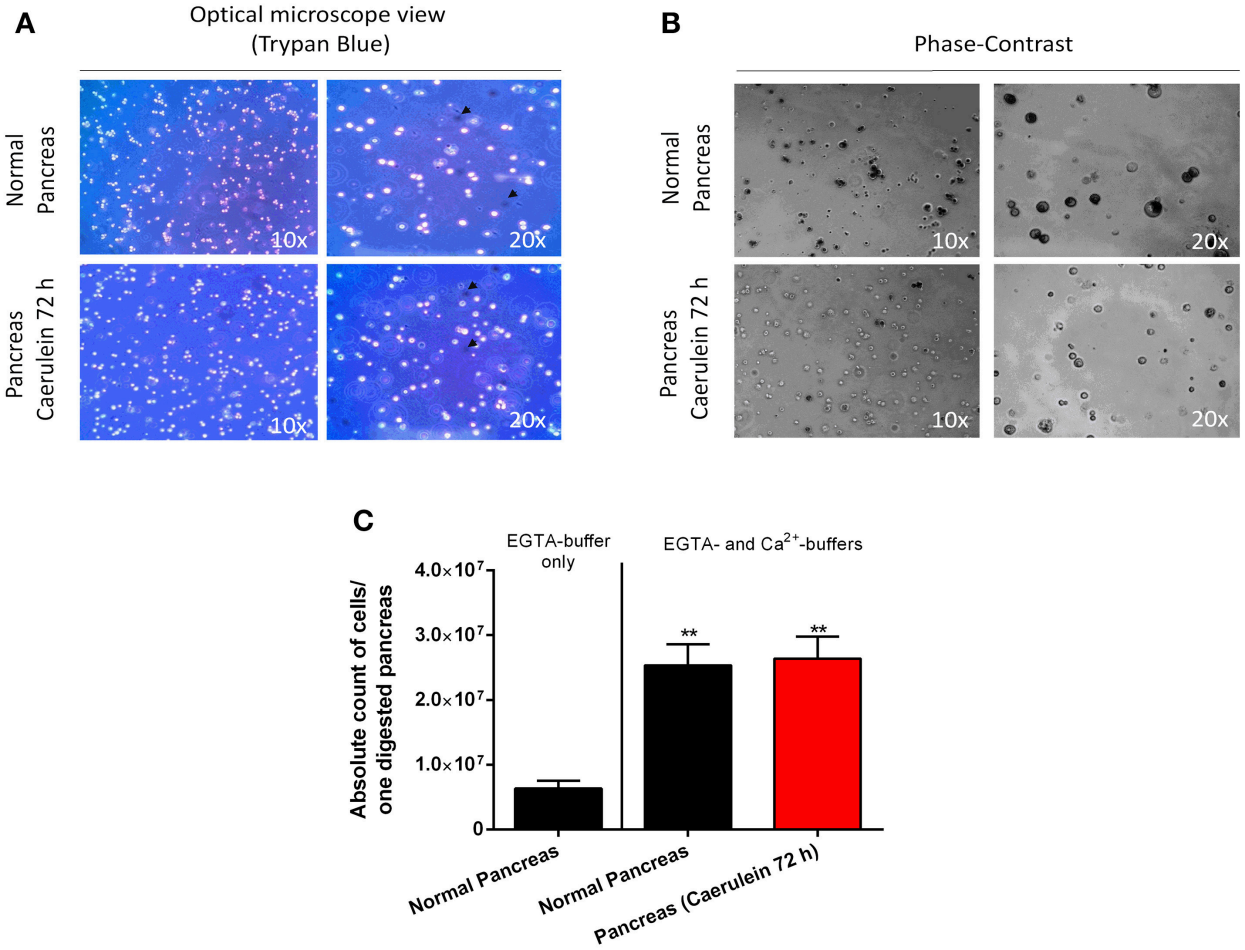
Assessment of cell viability, morphology, and count. **(A)** Cells from normal and caerulein-treated pancreas were mixed with trypan bleu dye (1:2) directly after digestion, then put in a Petri dish and visualized under an optical microscope (view 10 x and 20 x). Dead cells incorporated trypan blue and appeared dark (black arrows). **(B)** Phase-contrast images were taken for cells isolated from normal and caerulein-treated pancreas (view 10 x and 20 x). Cells from normal pancreas appeared larger and darker. **(C)** Absolute cell count was performed on 20 μL of digestion solution, using an automated hemocytometer. The EGTA-buffer only condition was shown to illustrate the low number of cells obtained when only this solution was used. Results were presented as mean ± SEM (EGTA-buffer only: *n* = 3; normal pancreas with EGTA- and Ca^2+^-buffers: *n* = 16; caerulein-treated pancreas 72 h with EGTA- and Ca^2+^-buffers: *n* = 17). Statistical significance was determined using a *t*-student test and *p*-values were considered statistically significant when *P* < 0.05 (^**^P < 0.01, compared to EGTA-buffer only).

Interestingly, the digestion protocol described here is applicable on both healthy and injured pancreas and may give rise to ~70-to-85% of viable cells. As depicted in Figure 3A, cells from healthy and caerulein-treated pancreas are well-dissociated and most did not incorporate trypan blue dye. Phase-contrast microscope images revealed that cells isolated from control pancreas appear larger and darker compared to those isolated from the caerulein-treated pancreas for 72 h (Figure 3B). This dark appearance could be attributed to the presence of zymogen vesicles that are lost by degranulation in injured acini (Cosen-Binker and Gaisano, 2007). However, although the dark aspect of acinar cells was also lost after 8 h of caerulein treatment, the size of the cells remained unchanged (Figure S2A). The average cell count gave 25 000 000 cells per adult pancreas in both normal and acute inflammatory conditions (Figure 3C and Figure S2B). Therefore, the protocol is efficient and reproducible, allowing the isolation of a high number of single-dissociated cells from healthy and injured pancreas.

### Sorting by flow cytometry allows the isolation of a given pancreatic cell type and the extraction of high quality rna

**Figure 4:**
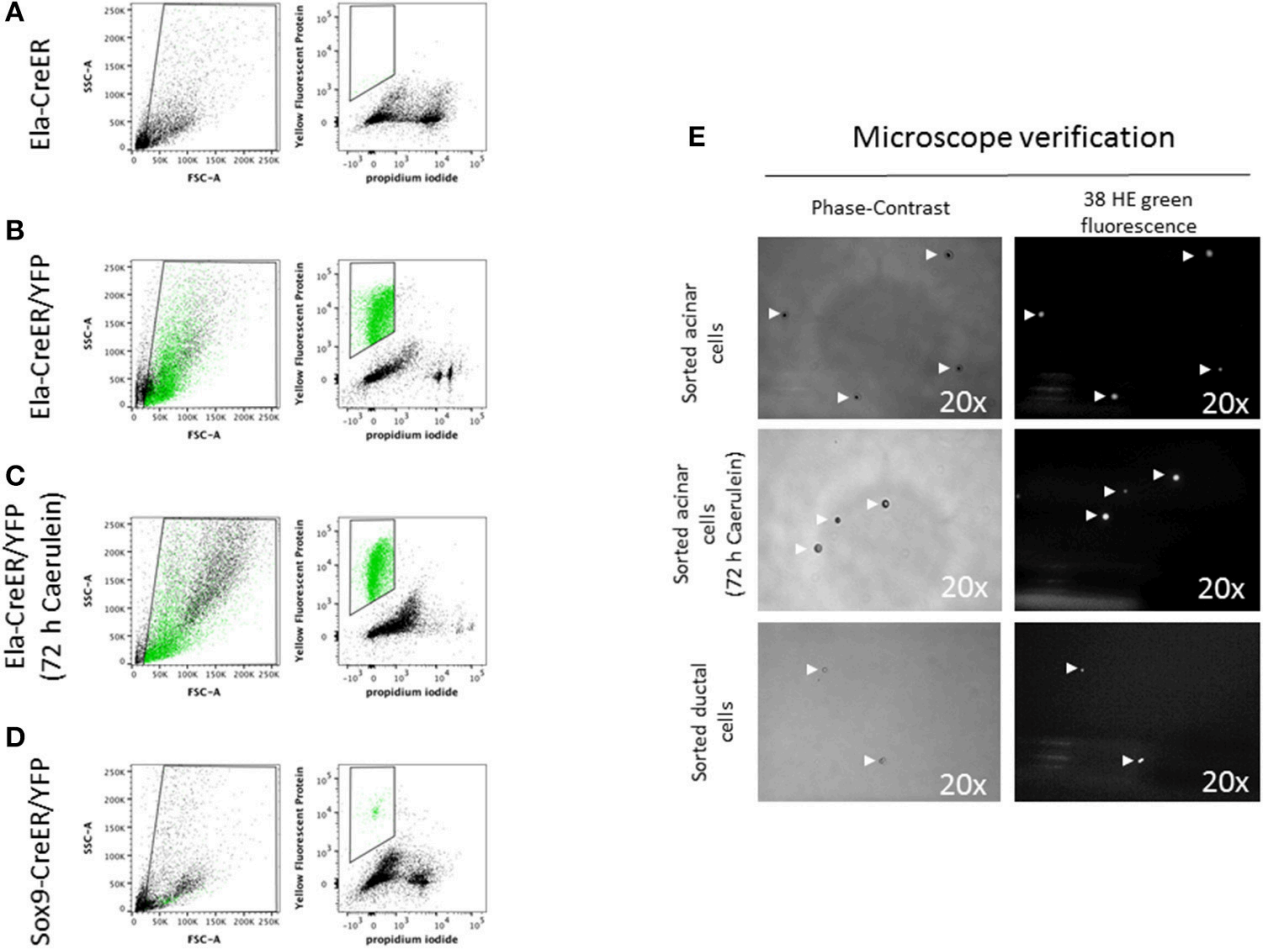
Flow cytometry sorting of YFP-positive acinar and ductal cells**. (A–D)** In the left panels, the designed window excludes debris and selects events that were distributed according to their internal content (e.g., granularity) and size on the SSC-A and FSC-A axes, respectively. In the right panels, events were represented according to their level of YFP and PI staining. YFP-positive events were marked in green. **(A)** The sorting gate was empty as the mouse used has a non-YFP Ela-CreER genotype. **(B,C)** Acinar cells from Ela-CreER/YFP mice, treated or not with caerulein, appeared in green within the sorting gate. The SSC-A/FSC-A panels indicated that untreated acinar cells possessed a somehow higher level of SSC-A compared to caerulein-treated cells. This was perhaps due to zymogene vesicles degranulation in the caerulein-treated group. **(D)** Ductal cells from Sox9-CreER/YFP mice appeared within the sorting window, but the number of gated cells was ~6-fold lower compared to sorted acinar cells. Additionally, as shown by the SSC-A/FSC-A panel, ductal cells exhibited a lower level of SSC-A compared to untreated and caerulein-treated acinar cells, which could reflect the less complex intracellular architecture of ductal cells. **(E)** After flow cytometry, a small volume of the sorting solution was subjected to microscope analysis. To check the morphology of the cells, images were taken using a phase-contrast objective (view 20 x). Indeed, ductal cells diameter appeared smaller than that of acinar cells. To verify that the sorted cells were YFP-positive, the emitted fluorescence was collected using a 38 HE green fluorescence channel (excitation line 470/40 nm, collection with Band-Pass filter 525/50 nm) (view 20 x).

Previous studies have detailed protocols to isolate pancreatic endocrine and mesenchymal cells using labeling with specific antibodies and flow cytometry sorting (Dorrell et al., 2011; Epshtein et al., 2017). However, the quality of extracted RNA from sorted cells has not been clearly reported. Genetic labeling by Cre-mediated excision of loxP-stop-loxP cassettes located upstream of genes encoding fluorescent proteins has become an unavoidable tool, not only for cell lineage tracing in developmental biology studies (Lemaigre, 2015), but also to isolate a specific set of cells for further in-depth experiments. In our study, we chose the Ela-CreER/YFP and Sox9-CreER/YFP models, as Cre-mediated recombination has been shown to be restricted in these models to acinar and ductal cells, respectively (Desai et al., 2007; Kopp et al., 2011). Compared to other protocols, the use of pancreas-specific Cre driver lines and fluorescent proteins is advantageous, as it permits the sorting of cells directly after pancreas digestion, reduces the time of cell manipulation and decreases cell loss by additional washing/centrifugation steps during antibody labeling. Before starting sorting on Ela- or Sox9-CreER/YFP mice, we used a control non-YFP mouse to set the levels of auto-fluorescence and avoid the gating of contaminating cells (Figure 4A). Acinar and ductal cells from Ela- and Sox9-CreER/YFP appeared as a single population high in YFP and negative for PI staining (Figures 4B–D and Figure S2C). The total number of isolated acinar cells, treated with or without caerulein, was ~6-fold higher than ductal cells (Figures 4B–D and Figure S2C). After flow cytometry, we visualized the sorted solution under a fluorescence microscope to ensure that it contains YFP-positive cells with good morphology. As depicted in Figure 4E, sorted acinar and ductal cells exhibited a round shape in bright field and emitted fluorescence that was detected by the 38 HE green fluorescence channel.

**Figure 5:**
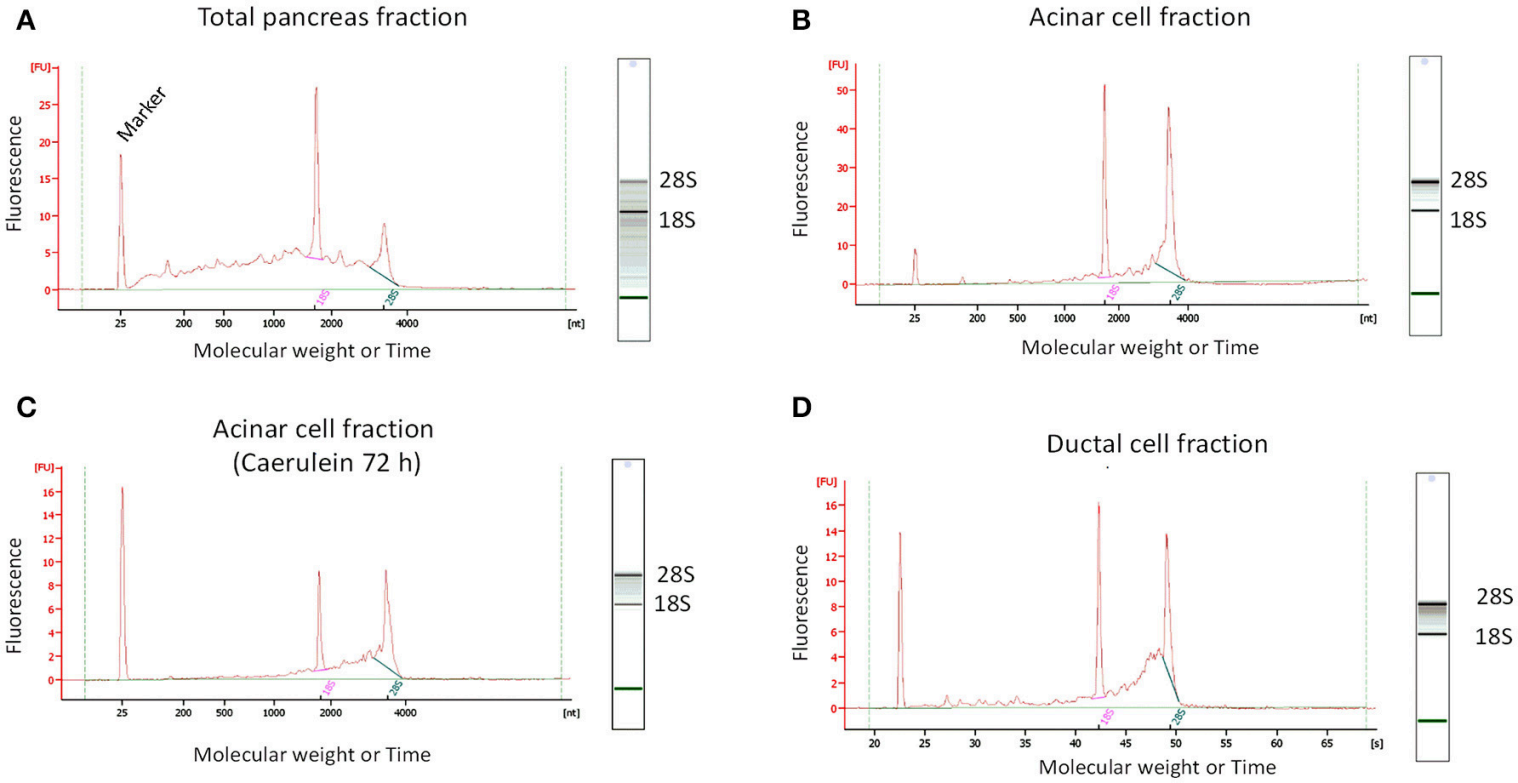
Determination of RNA quality by micro-electrophoresis. For standardized RNA quality control, 1 μL of total extracted RNA was added to each well in a twelve-well microfluidic chip. The wells were filled with gel containing an intercalating dye that allows RNA detection by a laser-induced fluorescence method. The results were then visualized as gel-like tracks and electropherograms (Y-axis: Fluorescence intensity and X-axis: Molecular weight or Time). **(A)** RNA from total pancreatic fraction was of poor quality, as electropherogram showed an elevated baseline (above the green line) and the gel-like track revealed the presence of a smear and low 28S rRNA band intensity. **(B–D)** The quality of RNA from sorted acinar and ductal fractions was significantly higher compared to total pancreas fraction. Indeed, electropherograms were tangent to the green line and the gel-like tracks appear to be smear-free.

After RNA extraction, we observed that the quality of RNA obtained from total pancreatic digestion before cell sorting was poor as evidenced by the presence of a smear throughout the gel-like track and low intensity of the 28S rRNA band (Figure 5A). By contrast, gel-like tracks corresponding to RNA extracted from sorted acinar and ductal cells indicated higher RNA quality, with the 28S rRNA band two times more intense than the 18S rRNA band (Figures 5B–D). The electropherogram provides interesting information about the quality of RNA. Indeed, an elevated baseline (above the green line) and a very low height in the 28S rRNA peak are strong indicators of RNA degradation (Schroeder et al., 2006). Based on these features, the bio-analyzer attributes for each sample a RIN value between 1 (completely degraded) and 10 (intact). Usually, RNA samples with a RIN between 6.9 and 9.5 are suitable for gene expression experiments. Interestingly, electropherograms of acinar and ductal cell fractions were tangent to the green line and presented less parasite peaks compared with the total pancreas fraction (Figure 5). The height of the 28S rRNA peak was slightly lower compared with 18S rRNA in sorted acinar and ductal fractions, indicating a weak level of degradation. However, the electropherogram profiles for both acinar and ductal fractions are typical to samples with a RIN of 8 (Schroeder et al., 2006).

We found that 150 000 sorted acinar cells were sufficient to obtain ~3 μg of total RNA with a RIN value of 8.5. Pancreatitis is usually associated with edema and fibrosis (Sparmann et al., 1997), which could limit the extraction of high quality RNA. An important finding of this study is that the quality of RNA from caerulein-treated mice for 8 and 72 h, respectively, was similar to that of untreated mice (Table 3). However, we found a time-dependent decrease in RNA quantity in caerulein-treated groups compared to control. For ductal cells, we sorted ~25 000 cells per pancreas and extracted ~350 ng of RNA with a RIN value of 7.4 (Table 3). In fact, we observed a proportional relationship between the number of sorted cells and the quality of RNA extracted from these cells. Thus, when two pancreata were digested separately and then sorted in one tube, the RIN value of RNA extracted from ductal cells (55 000 cells) climbed to 8 (data not shown). Together, the data indicate that flow cytometry sorting allows work on a population of live acinar or ductal cells free from extracellular matrix, fragmented cell membranes, and other contaminating material favoring the extraction of high quality RNA. The approach could be used to isolate cell subtypes from other organs. Table 4 lists the models in which the DIE-RNA protocol was tested by our laboratory.

**Table 3:** Quantity and quality of the extracted RNA.

**Cell fraction**	**Absolute RNA amount (ng)**	**RNA amount per Cell (pg)**	**RIN**
Total pancreas	50000 ± 7400	16.6 ± 2.4	4.8 ± 1.1
Acinar	3000 ± 200	16 ± 1.3	8.6 ± 0.17^**^
Acinar (8 h Caerulein)	2750 ± 396	11.5 ± 0.99	8.1 ± 0.1^*^
Acinar (72 h Caerulein)	886 ± 112	8 ± 1	8.4 ± 0.09^**^
Ductal	380 ± 20	12.6 ± 0.8	7.4 ± 0.46^*^

For RNA from the whole pancreas, the extraction was performed on a small volume of the entire digestion solution (~ 3,500,000 cells), before FACS sorting. For the acinar fraction treated or not with caerulein, the extraction was performed on an average number of 150,000 sorted cells. For the ductal fraction, the number was reduced to 25,000 sorted cells per pancreas. Results are presented as mean ± SEM (total pancreas: n = 3; acinar fraction: n = 7; caerulein-treated acinar fraction 8 h: n = 3; caerulein-treated acinar fraction 72 h: n = 11; ductal fraction: n = 3). Statistical significance was measured using a t-student test and p-values were considered statistically significant when P < 0.05 (

* *P < 0.05*,

** *P < 0.01, compared to total pancreas fraction)*.

**Table 4:** GEMM in which the DIE-RNA protocol was tested.

**GEMM model**	**Organ**	**Exp. condition**	**Isolated cells**
Ela-CreER/YFP	Pancreas	Normal and pancreatitis	Acinar cells
Ela-CreER-LSLKRAS^G12D^/YFP	Pancreas	Normal and pancreatitis	Acinar cells
Sox9-CreER/YFP	Pancreas	Normal	Ductal cells
Opn-CreER/YFP	Liver	Normal	Cholangiocytes
Sox9-GFP	Liver	Normal	Cholangiocytes

*Our laboratory applied the DIE-RNA protocol on different GEMMs to isolate cells and extract RNA from different tissues including pancreas and liver. Experiments on osteopontin (Opn)-CreER/YFP and Sox9-GFP were realized by M. Di-Luoffo (Personal communication)*.

### Isolated acinar and ductal cells are free from significant contamination by other cell types and exhibit the expected biological responses

Next, we investigated whether the sorted fractions were enriched in acinar and ductal cells. For this purpose, a reverse transcriptase reaction on 100 ng of extracted RNA from acinar or ductal cells was performed using the high-capacity cDNA reverse transcription kit. Real-time quantitative PCR (qPCR) analysis was then undertaken on the neo-synthetized cDNA with the KAPA SYBR FAST mix. The relative expression of specific acinar and ductal markers was normalized to housekeeping genes (*Actb, Gapdh, Rpl4*, and *Rpl19*) and calculated using the ΔΔCt method. The primers used are listed in Table S1.

**Figure 6:**
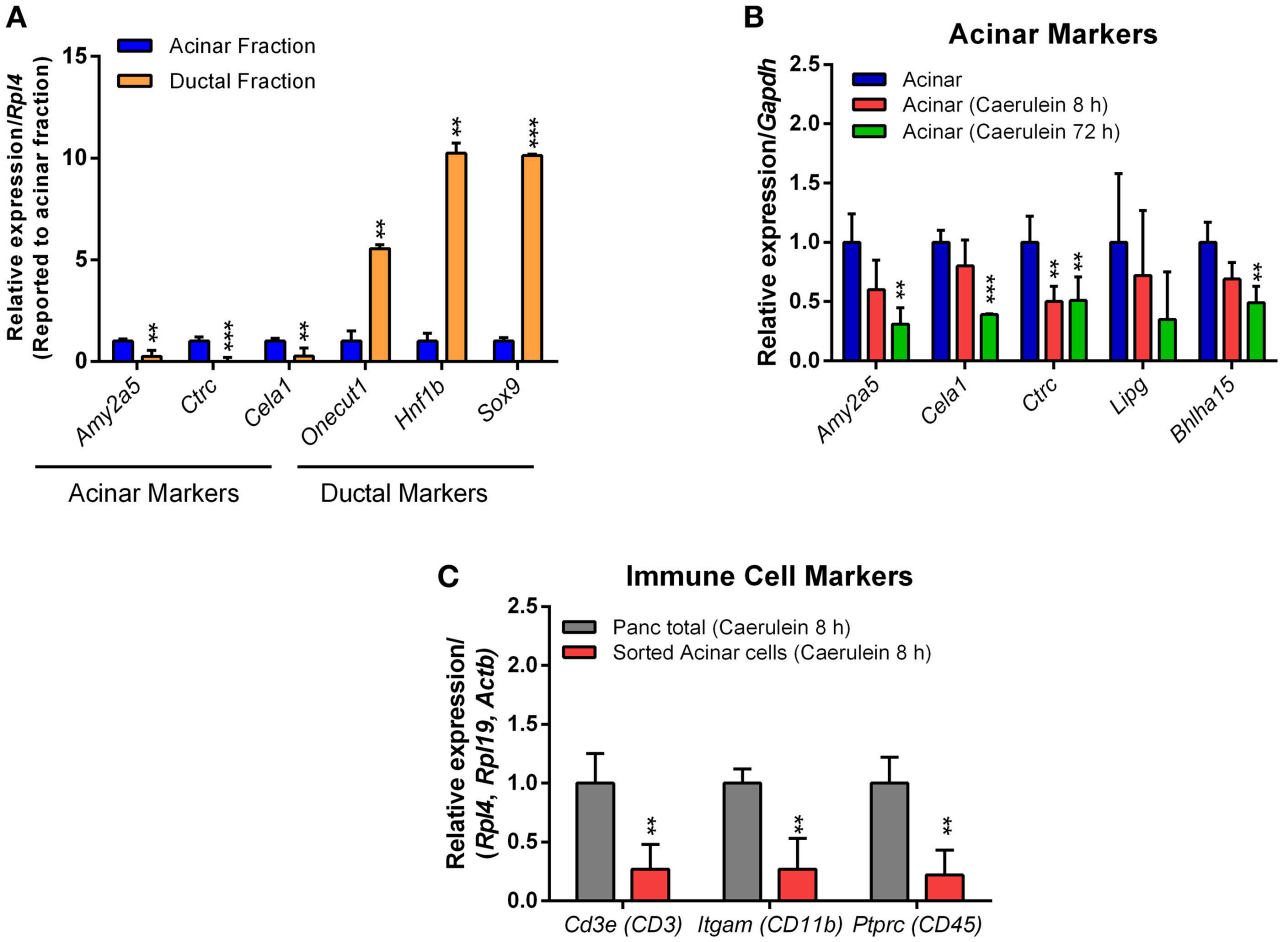
Expression of acinar, ductal, and immune cell markers by RTqPCR. The qPCR reaction was carried out in a final volume of 10 μL, containing: 5 μL of KAPA SYBR FAST mix, 1 μL of primers (10 μM each), 1 μL of cDNA and 3 μL of nuclease-free water. **(A)** For acinar cells, four different samples sorted from the pancreas of Ela-CreER/YFP mice were analyzed in duplicate on a 96-well opaque PCR plate. For ductal cells, one sample obtained from the pancreas of Sox9-CreER/YFP mouse and one other sample obtained from the combination of two pancreas of Sox9-CreER/YFP mice were analyzed in duplicate on the same PCR plate. The expression of target genes was normalized to the reference gene *Rpl4*. **(B)** Sorted acinar cells obtained from four untreated Ela-CreER/YFP mice and acinar cells sorted from three caerulein-treated Ela-CreER/YFP mice at 8 and 72 h were analyzed in duplicate on the same PCR plate. The expression of target genes was normalized to the reference gene *Gapdh*. **(C)** Measurement of immune cell markers was performed in duplicate on three total pancreas fractions before sorting and three sorted acinar fractions, after 8 h of caerulein treatment. Given the variability between total pancreas and sorted acinar cell fractions, the expression of target genes was normalized to three housekeeping genes (*Actb, Rpl4*, and *Rpl19*). Results were presented as mean ± SEM (Untreated acinar fraction: *n* = 4; caerulein-treated total pancreas fraction 8 h: *n* = 3; caerulein-treated acinar fraction 8 h: *n* = 3; caerulein-treated acinar fraction 72 h: *n* = 3 and ductal fraction: *n* = 3). Statistical significance was tested using a *t*-student test and *p*-values were considered statistically significant when *P* < 0.05 (^**^P < 0.01, ^***^P < 0.001, compared to untreated acinar fraction or caerulein-treated total pancreas fraction 8 h).

As anticipated, high expression of acinar markers such as *Amy2a5, Ctrc*, and *Cela1* was observed in acinar RNA preparations, compared with ductal markers (Figure 6A). Conversely, the expression of ductal markers like *Sox9, Hnf1b*, and *Onecut1* was much higher in the ductal RNA preparations (Figure 6A). We determined how caerulein treatment affects the expression profile of acinar markers in sorted acinar cells from caerulein-treated Ela-CreER/YFP mice. After 8 h of caerulein injections, the expression of acinar markers, with the exception of *Ctrc*, tended to decrease compared with control but was not statistically significant (Figure 6B). In contrast, the expression of *Amy2a5, Ctrc, Cela1*, and *Bhlha15* was strongly decreased at 72 h of caerulein treatment (Figure 6B). This result was expected, as early acinar-to-ductal metaplasia induced by caerulein were associated with a decrease in the expression of acinar markers (Prévot et al., 2012; Dey et al., 2014). As caerulein-induced pancreatitis is associated with infiltration of immune cells, we tested whether the sorted acinar cells were contaminated with this population. We measured the expression of well-known immune cell markers [*CD3e* (CD3), *Itgam* (CD11b), and *Ptprc* (CD45)] by RTqPCR and compared their expression level in the total pancreas fraction before sorting and in sorted acinar cells after 8 h of caerulein treatment. We demonstrated that the expression of *CD3e, Itgam*, and *Ptprc* was 4-to-5-fold lower in sorted acinar cells (Figure 6C). The low levels of *CD3e, Itgam*, and *Ptprc* observed in sorted acinar cells, could be due to the ability of acini to express these markers at the mRNA level in response to injury (De Dios et al., 2005). Therefore, together the data presented suggest that isolated acinar and ductal cell fractions were of high purity.

## Conclusion, strengths, and limitations

In summary, the protocol described allows a rapid and efficient digestion of pancreas into single-dissociated cells, the sorting of pure acinar and ductal fractions and the preparation of high quality RNA suitable for sensitive gene expression experiments such as RNA sequencing and microarray. A significant advantage of the protocol is that it can also be applied without modifications to injured pancreas, even in the presence of an oncogenic insult of KRAS^G12D^ mutation (data not shown). The protocol is short and reproducible (*n* = 40 RNA preparations) and can be carried out on other organs, such as liver. In addition, Ela- or Sox9-CreER/YFP mice are models currently available in many laboratories. One of the limitations of this study is the low number of sorted cells for some cellular subsets, such as pancreatic ductal cells. Therefore, in this case, the combination of cells from two or more pancreata could be necessary. We note that 50 000 sorted cells is the minimum threshold that should be reached to obtain a sufficient quantity of high quality RNA. Another limitation is the efficiency of Cre recombination that may vary according to responsiveness of mice to TAM and the model of Cre driver lines. A last limitation results from the time that flows (~2 h) between the moment when the mice are sacrificed and the cells are lysed. During this time, the cells are under stress resulting from the isolation procedure. This stress could lead to changes in the expression levels of transcripts. When analyzing results, the researcher must be aware of this possibility, although it is expected that these changes are identical between samples being compared. Overall, the present protocol facilitates the isolation of different cellular subsets for comparative analyses. This is important to detect limited but significant biological responses that could be drowned or be undetectable when measurements are performed on whole pancreatic tissue.

## Author contributions

MA and PJ designed this work; MA performed all experiments, drafted, and revised the manuscript; MA and ND performed flow cytometry experiments; MA, ND, and PJ interpreted the data; PJ critically revised the paper; PJ supervised this work. All authors approve the final version of this manuscript.

### Conflict of interest statement

The authors declare that the research was conducted in the absence of any commercial or financial relationships that could be construed as a potential conflict of interest.

## Supplementary material

Table S1 List of primers used for RTqPCR analysis.

Figure S1 Detailed graphical illustration of pancreas digestion protocol.

Figure S2 Application of DIE-RNA protocol on caerulein-treated mice for 8 h. **(A)** Phase-contrast images were taken for cells isolated from caerulein-treated pancreas (view 10 x and 20 x). **(B)** Absolute cell count was performed on 20 μL of digestion solution, using an automated hemocytometer. The EGTA-buffer only condition is shown to illustrate the low number of cells obtained when only this solution is used. Results were presented as mean ± SEM (EGTA-buffer only: *n* = 3; caerulein-treated pancreas 8 h with EGTA- and Ca^2±^-buffers: *n* = 3). Statistical significance was determined using a *t*-student test and *p*-values were considered statistically significant when *P* < 0.05 (^**^*P* < 0.01, ^***^*P* < 0.001 compared to EGTA-buffer only) **(C)** Flow cytometry sorting of YFP-positive events.

## Abbreviations

Actb: Beta Actin gene
Amy2a5: Amylase gene
Bhlha15: Mist1 gene
Ca^2+^: Calcium
Cd3e: CD3 gene
Cela1: Elastase gene
Col: Collagenase
cDNA: Complementary deoxyribonucleic acid
Ctrc: Chymotrypsin gene
DNA: Deoxyribonucleic acid
DPBS: Dulbecco's phosphate-buffered saline
EGTA: Ethylene glycol-bis(beta-aminoethyl ether)-N,N,N',N'-tetraacetic acid
Ela: Elastase
FSC: Forward scatter
Gapdh: Glyceraldehyde-3-phosphate dehydrogenase
GEMM: Genetically engineered mouse models
GFP: Green fluorescent protein
Hnf: Hepatocyte nuclear factor
Itgam: CD11b gene
Lipg: Lipase gene
Opn: Osteopontin
PanIN: Pancreatic intraepithelial neoplasia
PCR: Polymerase chain reaction
PI: propidium iodide
RNA: Ribonucleic acid
rRNA: Ribosomal RNA
RIN: RNA integrity number
Rpl4: Ribosomal protein L4
Rpl19: Ribosomal protein L19
Sox9: SRY (sex determining region Y)-box 9
SSC: Side scatter
TAM: Tamoxifen
YFP: Yellow fluorescent protein.
